# Alternate virtual populations elucidate the type I interferon signature predictive of the response to rituximab in rheumatoid arthritis

**DOI:** 10.1186/1471-2105-14-221

**Published:** 2013-07-10

**Authors:** Brian J Schmidt, Fergal P Casey, Thomas Paterson, Jason R Chan

**Affiliations:** 1 Entelos Holding Corporation, 2121 South El Camino Real, Suite 600, San Mateo, CA 94403, USA

**Keywords:** Drug development, Mechanistic modeling, Biosimulation, Patient population, Biomarker, Pharmaceutical research & development, Personalized medicine

## Abstract

**Background:**

Mechanistic biosimulation can be used in drug development to form testable hypotheses, develop predictions of efficacy before clinical trial results are available, and elucidate clinical response to therapy. However, there is a lack of tools to simultaneously (1) calibrate the prevalence of mechanistically distinct, large sets of virtual patients so their simulated responses statistically match phenotypic variability reported in published clinical trial outcomes, and (2) explore alternate hypotheses of those prevalence weightings to reflect underlying uncertainty in population biology. Here, we report the development of an algorithm, MAPEL (Mechanistic Axes Population Ensemble Linkage), which utilizes a mechanistically-based weighting method to match clinical trial statistics. MAPEL is the first algorithm for developing weighted virtual populations based on biosimulation results that enables the rapid development of an ensemble of alternate virtual population hypotheses, each validated by a composite goodness-of-fit criterion.

**Results:**

Virtual patient cohort mechanistic biosimulation results were successfully calibrated with an acceptable composite goodness-of-fit to clinical populations across multiple therapeutic interventions. The resulting virtual populations were employed to investigate the mechanistic underpinnings of variations in the response to rituximab. A comparison between virtual populations with a strong or weak American College of Rheumatology (ACR) score in response to rituximab suggested that interferon β (IFNβ) was an important mechanistic contributor to the disease state, a signature that has previously been identified though the underlying mechanisms remain unclear. Sensitivity analysis elucidated key anti-inflammatory properties of IFNβ that modulated the pathophysiologic state, consistent with the observed prognostic correlation of baseline type I interferon measurements with clinical response. Specifically, the effects of IFNβ on proliferation of fibroblast-like synoviocytes and interleukin-10 synthesis in macrophages each partially counteract reductions in synovial inflammation imparted by rituximab. A multianalyte biomarker panel predictive for virtual population therapeutic responses suggested population dependencies on B cell-dependent mediators as well as additional markers implicating fibroblast-like synoviocytes.

**Conclusions:**

The results illustrate how the MAPEL algorithm can leverage knowledge of cellular and molecular function through biosimulation to propose clear mechanistic hypotheses for differences in clinical populations. Furthermore, MAPEL facilitates the development of multianalyte biomarkers prognostic of patient responses in silico.

## Background

Rheumatoid arthritis (RA) is a debilitating, progressive disease that affects approximately 1% of the adult population [1]. Clinically, RA is a symmetric polyarticular disease that is characterized by swollen and tender joints, the presence of circulating factors such as elevated C-reactive protein and rheumatoid factor, and the degradation of cartilage and bone [1,2]. In addition to potential disability, rheumatoid arthritis is also a risk factor for cardiovascular disease and mortality.

Mechanistically, joint function is degraded by infiltration of the synovial lining by cells of the innate and adaptive immune system. Many cellular players are involved in initiating and sustaining the inflammatory response, including fibroblast-like synoviocytes, lymphocytes, monocytes, and others [3]. The paracrine feedback of pro- and anti-inflammatory mediators makes deciphering causation difficult. Early attempts to characterize RA supported several distinct causative hypotheses, which suggested that specific soluble factors, T cells, or B cells were the dominant agent. It is now accepted the etiology is complex and that many innate and adaptive immune cell types have an important mechanistic role [1].

Patients exhibit a spectrum of responses to clinical therapies for RA. To assist in the assessment of therapeutic efficacy, the American College of Rheumatology has defined standards that have been employed in clinical trials to evaluate the relative improvement in the clinical, inflammation-dependent manifestation of RA with therapy [4]. Notably, only about 50% of patients with early RA achieve a mild American College of Rheumatology 20 (ACR20) response to methotrexate, a first line therapy for treatment [5]. Some patients that do not respond well to methotrexate respond well to the combination of methotrexate and a biologic drug targeting tumor necrosis factor α (TNF). For example, about 60% of patients exhibited a mild ACR20 response and 25% a strong ACR70 response when treated with infliximab [6]. In lieu of a single therapeutic panacea, a variety of marketed biological therapies target alternate cytokines and cellular antigens including: interleukin-6 receptor (IL6R, tocilizumab), interleukin-1 (IL-1, anakinra), cytotoxic T-lymphocyte antigen 4 (CTLA4, abatacept), and cluster of differentiation 20 (CD20, rituximab). The emergence of multiple, mechanistically distinct targets has been suggested to reflect the existence of unique states of the network underlying RA [7] and/or heterogeneity in the pathogenesis of the disease. Indeed, despite these additional therapeutics, there remain patients that do not respond well to treatment.

The heterogeneity in disease states presents a challenge for the effective development of new therapies by the pharmaceutical industry. Economic analysis suggests that the pharmaceutical industry tends to pursue targets in therapeutic areas where the risk of failure is high [8]. When there is an opportunity to be first-in-class and with a clear unmet medical need, the economic reward is great due in substantial part to a lack of competition [8]. In RA, the presence of therapies that work well in some patients, such as anti-TNF agents, increase the risk to drug development since it is not clear whether a new drug will be more effective in an underserved, identifiable portion of the population. Although meta-analysis techniques have demonstrated utility to halt the development of generally unpromising compounds early in clinical development [9], it is difficult to extrapolate clinical benefit for select patient populations from preclinical models. Phenotype-driven mechanistic biosimulation can be employed to develop testable hypotheses governing efficacy, evaluate therapeutic targets in silico, address alternate scenarios of efficacy, and identify predictive biomarkers [10].

Methods for population PK/PD modeling are relatively mature (for a discussion, refer to [11]). In contrast, statistical calibration of large mechanistic biosimulation results to analyze trial efficacy at the population level has been proposed [12], but further development and implementation of relevant methods is still needed. These approaches are potentially of tremendous benefit to virtual study population selection. For example, characterization of virtual patient (VP) diversity has previously been employed in the context of selecting clinical trial endpoints and optimizing trial design [13]. The assignment of prevalence weights to individual VPs to statistically recreate clinical outcome distributions in a virtual population (VPop) has been previously validated in the context of type 2 diabetes [14]. In this study, dimensionality of the patient variability space was effectively reduced by employing principal component analysis (PCA), and the prevalence weight of individual VPs was adjusted to create a VPop with optimal agreement with data from NHANES III. Studies in drug-induced liver injury (DILI) have used parameter distribution and drug response fitting approaches to guide the development of each VP in a single population, which has been called a “SimPops” approach [15,16]. Despite the successes of methods to develop VPops, there are challenges that limit their general applicability. First, a method for creating alternate VPops that also agree with trial data is desirable. This would more directly facilitate a comparison of best and worst-case scenarios for the improvement with therapy at the population level. Second, databases such as NHANES III contain multiple measures for individual patients, including the response to meaningful perturbations such as an oral glucose tolerance test, that facilitate dimensionality reduction by PCA. Other therapeutic areas may not have a comparable resource to assist in the multivariate analysis of patients. For example, in RA, trials of new therapies often must run for six months to one year to assess therapeutic efficacy. Additionally, it is more difficult to measure the concentrations of critical mediators in the synovial tissue. Although relevant data is available in the literature, comprehensive multivariate measures for each patient in clinical trials are not.

We report here the development of a novel algorithm, MAPEL (Mechanistic Axes Population Ensemble Linkage), for developing an ensemble of statistically-calibrated VPops that enables the development and empowers the interpretation of biomarkers from biosimulation data. The algorithm has been developed specifically to utilize data embedded in the mechanistic parameters that characterize the VPs that underlie the biosimulation. We apply the algorithm to a previously validated model of a rheumatic joint [17,18]. The ensemble of VPops thus developed agreed well with a reported baseline type I interferon cytokine signature that distinguishes patients that respond well to rituximab [19]. We then employed the ensemble of VPops and the biosimulation platform as tools to probe the mechanistic basis of the reported biomarker in silico since these mechanisms remain unclear in the literature.

## Methods

### Biosimulation

**Figure 1:**
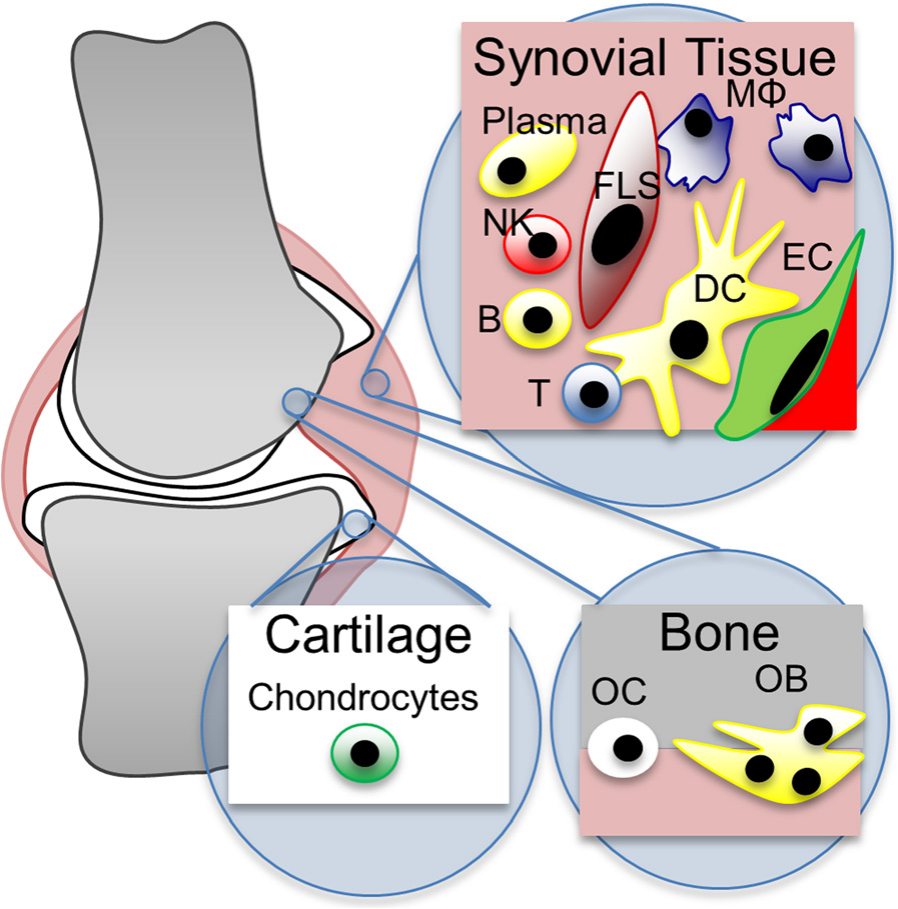
**Scope of the biology represented in the biosimulation platform.** In the synovial tissue, the activity of fibroblast-like synoviocytes (FLS), B cells (B), plasma cells, natural killer cells (NK), macrophages (MΦ), and endothelial cells (EC) are modelled. T cells (T) are modelled with distinct CD4 and CD8 T cell subpopulations. CD4 T cells are further divided into Th1, Th2, Th17, and Treg subpopulations, as well as a CD28^-^ subset that has lost the requirement for costimulation. Antigen presentation by dendritic cells (DCs) is also represented. Bone remodelling by osteoclasts (OC) and osteoblasts (OB) as well as cartilage remodelling by chondrocytes and MMPs is included.

Biosimulation of a rheumatic joint was conducted with the Entelos RA PhysioLab® platform. As reported previously [17,18], the biosimulation platform mechanistically links cellular and cytokine components of the pathophysiology across multiple compartments, including the synovial lining, cartilage, and bone to patient- level clinical measures. Fibroblast-like synoviocytes (FLS), endothelial cells, macrophages, B cells, plasma cells, CD4 Tcells (including Th1, Th2, Th17, CD4^+^CD28^-^, and Treg subsets), CD8 T cells, NK cells, chondrocytes, osteoclasts, and osteoblasts are represented, as depicted in Figure 1. The synthesis of soluble factors including autoantibodies, TNF, IFNγ, IFNβ, IL-1β, IL-6, and various matrix metalloproteinases (MMPs) is also represented. Mechanistically regulated cellular activities include recruitment, proliferation, apoptosis, activation, and differentiation. The represented clinical outputs include a continuous ACR-N score, joint space narrowing (JSN), and bone erosion score (BES), and are directly tied to reductions in the cellular infiltrate, rate of cartilage degradation, and rate of bone degradation, respectively. An expanded discussion of relevant modeling methodology for autoimmune disorders has been given previously [20].

### Virtual patient cohort

To facilitate the development of population-level statistics, a cohort of VPs was first developed for analysis. Cohort VPs vary from each other in their mechanistic axes coefficients, which map onto individual model parameters. In contrast to the development of a single reference VP with responses to match the mean behavior reported in clinical trials [18], the acceptable responses for a VP cohort are broader and span a larger range of responses observed in the clinic [17]. When creating the cohort, mechanistic variability in pathophysiology was introduced through variation in mechanistic axes selected on the bases of sensitivity and diversity. The aim was to capture the full spectrum of observed clinical responses to multiple therapies with distinct mechanisms of action. Once a few VPs were calibrated manually, additional VPs were created using a genetic algorithm on a computing cluster. To ensure reasonable pathologic characteristics of the VPs, the literature was reviewed for reported ranges for the density of the various inflammatory cells found in the synovium and cytokine concentrations reported in the serum (Additional file 1). The observed ranges were used to evaluate the validity of VPs as they were returned by the algorithm. Clinical responses to approved therapies for patients with inadequate responses to methotrexate were tested to ensure the full range of clinical response was observed in the VP cohort. Mechanistic axes coefficients and therapeutic response results for the virtual patient cohort are provided (included in Additional file 2).

### MAPEL algorithm

**Figure 2:**
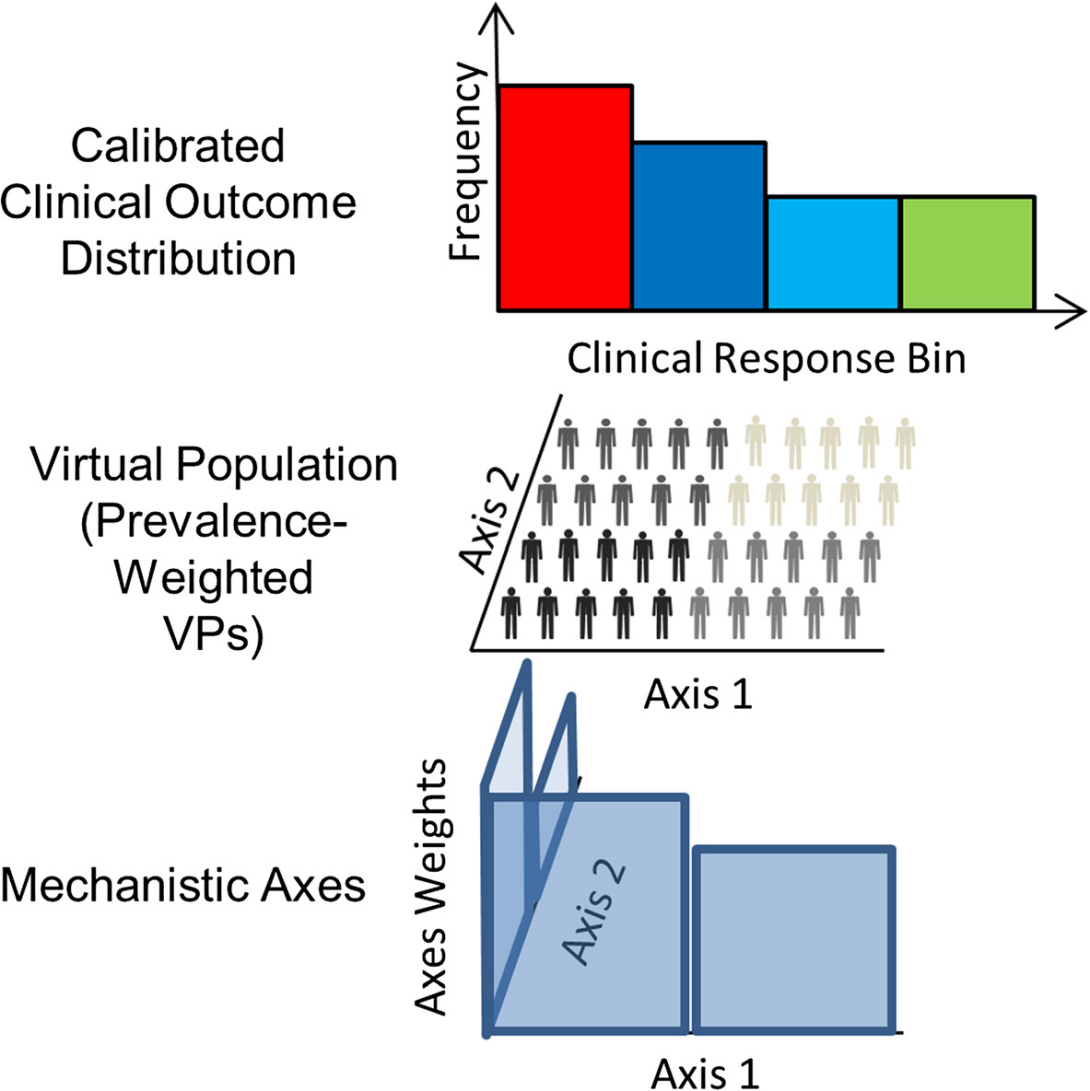
**Relation of mechanistic axes to measured population-level therapeutic responses.** The MAPEL algorithm for developing VPops links the mechanistic axes underlying biosimulation to clinical statistics through VPs. In the bottom panel, MAPEL assigns probability distributions directly to the mechanistic axes. A simple case of two axes is shown for clarity. In the middle panel, prevalence weights for VPs in the cohort are calculated. The prevalence weight is essentially a measure of the fraction of the total VPop that a given VP statistically represents. The prevalence weight of each VP is calculated from the axes weights assigned by MAPEL. VPs assigned a higher weight are depicted as darker colors. In the top panel, the VPop’s clinical response distribution is calculated. As described in the text, the Entelos RA Physiolab^®^ platform was re-run for each individual VP in the population for each therapy. Therefore, biosimulation results provide the response to therapies for each VP. These simulated responses to therapy were used in combination with the prevalence weights to calculate population-level responses to therapy. The calculation of a binned response distribution is shown, e.g. ACR20, 50, 70. In addition, weighted means and weighted standard deviations are also calculated, as detailed in the methods. In practice, MAPEL varies the axes weights until multiple clinical response distributions are in agreement with published trial statistics.

A new algorithm was developed using R [21] to efficiently create VPops that match clinical trial statistics from the large cohort of about 1,200 VPs, as illustrated conceptually in Figure 2. A brief summary of key concepts is presented in Table 1. Essentially, the MAPEL algorithm derives a prevalence weight for each VP in the cohort by assigning and optimizing probability distributions on each of the mechanistic axes. MAPEL then calculates population-level statistics from the appropriately-weighted individuals and their clinical responses. By contrast, a prevalence weight could be assigned to each VP directly based on model outputs, but this involves a much larger number of free parameters, about one per VP, and becomes computationally prohibitive as the number of VPs increases. The approach employed by MAPEL has the attractive characteristic that the mechanistic axes themselves are utilized for the optimization.

**Table 1:** Summary of key concepts

**Term**	**What is it and how is it used?**
Virtual Patient (VP)	Each VP has simulated clinical responses to multiple therapeutic interventions and associated pre-intervention measures, such as cell counts and synovial cytokine concentrations. 1,206 VPs were developed.
Mechanistic axes	VPs are defined by where their biology lies on the mechanistic axes. The axes introduce heterogeneity into the VPs. Axes define different VPs. Fifty-one alternate mechanistic axes were selected on the bases of sensitivity and diversity.
Axes weights	MAPEL assigns probabilities along the axes that are used to calculate the prevalence weight of each VP. The details are discussed in the methods.
Prevalence weight	A prevalence weight is the frequency of a VP relative to other VPs. The prevalence weight is used to calculate trial statistics. For example, 1 of the 1,206 virtual patients might be assigned 1% of the total weight even though it only accounts for 0.1% of the VPs. This VP’s prevalence weight would be 0.01. Note that all VPs receive a prevalence weight, based on the axes weights, although the weights will not be equal.
Virtual Population (VPop)	A single VPop is defined by one full set of prevalence weights for the VPs, e.g. one set of 1,206 prevalence weights. When the prevalence weights are applied to the simulated trial outcomes for each VP, the resulting statistics better match trial populations.

**Figure 3:**
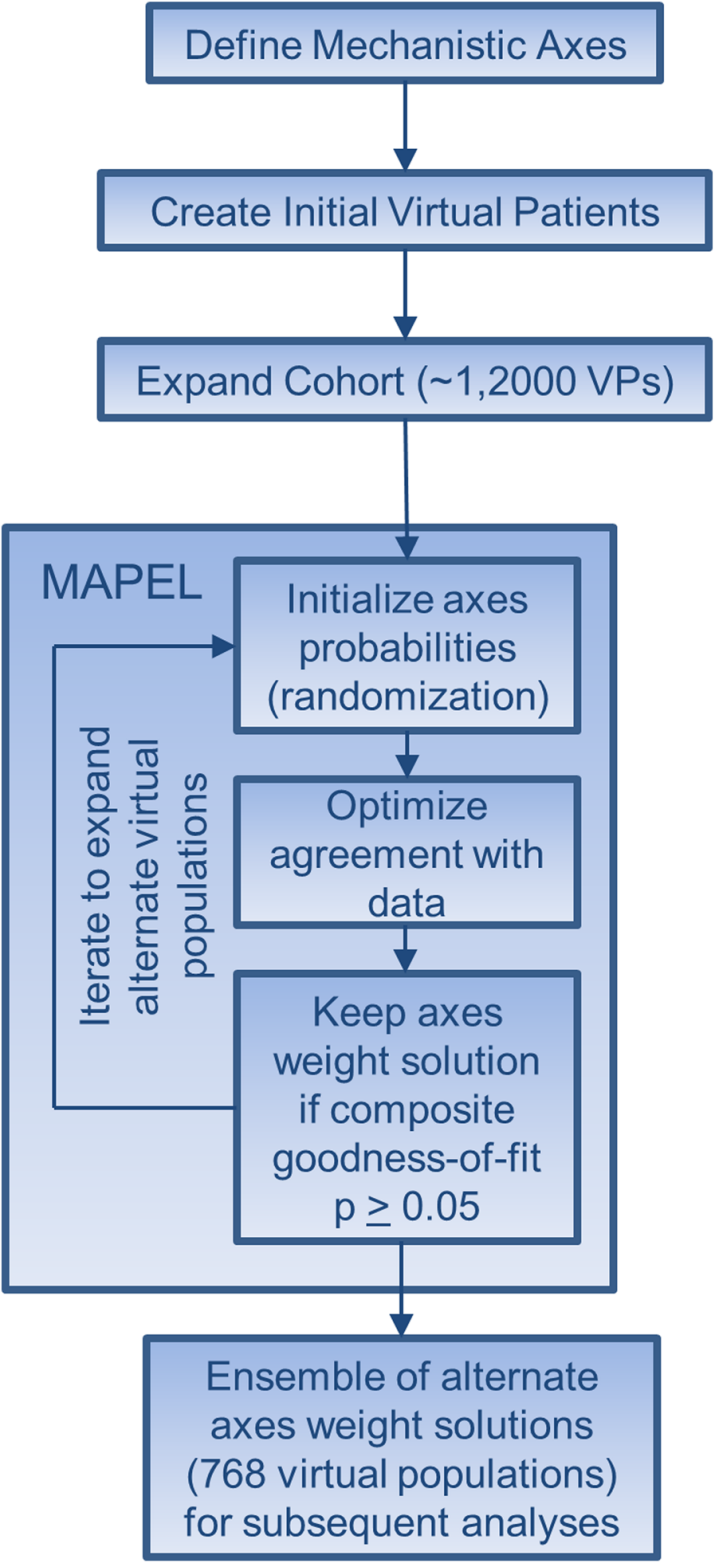
**Workflow for generating the ensemble of virtual populations.** MAPEL can be applied iteratively to create an ensemble of acceptable axes weight solutions, or an ensemble of VPops. The general workflow is shown for clarity. First, valid VPs that meet the acceptance criteria detailed in the methods were developed. In total, a cohort of 1,206 VPs was created by introducing diversity along 51 mechanistic axes using a genetic algorithm and screening for VPs with realistic pathophysiology at baseline and feasible responses to therapy. The cohort of VPs was then used with the aggregated clinical trial data to inform the MAPEL algorithm. Valid axes weight solutions that defined VPops were randomized and used as starting points for additional iterations to create alternate virtual populations. Ultimately, 768 alternate VPops were developed for subsequent analyses.

We chose to focus on clinical populations that did not respond adequately to methotrexate, since this population is of interest for the development of new therapeutics. When running MAPEL, a statistical comparison to published clinical trial data was performed on the basis of ACR responses to adalimumab, infliximab, rituximab, and tocilizumab, and clinical trial data was gathered from the references in Table 2. The details of the algorithm follow below, and a summary of the workflow is depicted in Figure 3.

**Table 2:** Clinical trials used to calibrate virtual populations with MAPEL

**Therapy**	**Trial in MTX-IR patients**
Rituximab, 1000 mg/kg, background MTX	[22]
Tocilizumab, 4 & 8 mg/kg, background MTX	LITHE [23], OPTION [24]
Anti-TNF (infliximab 3 & 10 mg/kg, adalimumab 40 mg/kg), background MTX	ATTRACT [6,25], ATTEST [26]

### MAPEL step 1: initialization

The mechanistic axes were split into two bins, representing the lower and upper fiftieth percentile of values from cohort VPs, to ensure equal representation in the high and low bins. During the initialization of the optimization, each bin was randomly assigned a weight. It should be noted that since the marginal probabilities along each axis sum to 1, there is only one free probability variable per mechanistic axis.

### MAPEL step 2: prevalence weight calculation from axes weights

Each VP’s normalized, prevalence weight relative to other VPs in the VPop can be calculated from the M mechanistic axes according to

(1) $$w_{i}=\prod_{j=1}^{M}\left(1_{C_{i,j}\in L_{j}}\left(1-p_{j}\right)+1_{C_{i,j}\in U_{j}}p_{j}\right)$$

where C_i,j_ is the value of axis j for VP i, L_j_ indicates the interval for the lower bin of axis j, U_j_ indicates the interval for the upper bin for axis j, p_j_ indicates the weight on the upper bin of axis j, and **1** is the indicator function. Additionally, a correlation can be introduced between axes k and l, and the prevalence weight calculation will allow for a bivariate normal distribution between the two axes. The correlation coefficient is then a parameter to be estimated along with individual axis probabilities. In this case, the prevalence weight for the i^th^ VP can then be calculated according to

(2) $$\begin{array}{l} w_{i}=\frac{\sigma_{l}\sigma_{k}}{\sqrt{\left|\Sigma \left(\rho \right)\right|{\left(2\pi \right)}^{2}}}\int_{a_{i,l}}^{b_{i,l}}\int_{a_{i,k}}^{b_{i,k}}e^{-\frac{1}{2}\left(\theta^{T}\Sigma {\left(\rho \right)}^{-1}\theta \right)}\mathrm{d\theta}\prod_{j\neq k,l}\\ \;\times \left(1_{C_{i,j}\in L_{j}}\left(1-p_{j}\right)+1_{C_{i,j}\in U_{j}}p_{j}\right) \end{array}$$

Here, axis k and l are correlated with correlation matrix Σ(ρ), that is a 2×2 matrix with ones on the diagonal and value ρ on the off diagonal. Also, θ is defined by:

(3) $$\theta =\left[\frac{C_{i,l}-\mu_{l}}{\sigma_{l}},\frac{C_{i,k}-\mu_{k}}{\sigma_{k}}\right]$$

This is the z-transformed mechanistic axis value for axis l and k. The integral term is for a strictly bivariate normal case in the present algorithm formulation (higher dimensional multivariate correlations were not explored). The limits of integration are determined by finding the z-value corresponding to the assumed bin probabilities in the present iteration by application of the inverse of normal univariate distribution function, Φ^-1^(p). The limits of integration depend on the bin for axis coefficient j for VP i. Note the lower and upper integration limits for VP i on axis j, a_i,j_ and b_i,j_, vary. If the axis coefficient falls into the lower bin (C_i,j_ ∈ L_j_):

(4) $$a_{i,j}=-\infty$$

Otherwise, for the higher bin (C_i,j_ ∈ U_j_):

(5) $$a_{i,j}=\Phi^{-1}\left(p_{i,j}\right)$$

The upper limit of integration also varies with the bin. For the lower bin:

(6) $$b_{i,j}=\Phi^{-1}\left(1-p_{i,j}\right)$$

For the higher bin:

(7) $$b_{i,j}=\infty$$

Note that the Gaussian term in formula (2) simplifies to a product of probabilities, p_j_ in the case when ρ is zero as we would expect. The preceding formulas are derived by applying a Gaussian bivariate *copula* to introduce a dependence between mechanistic axis l and k, while preserving the marginal distributions (for example, [27]).

### MAPEL step 3: response to therapy at the virtual population level

Once the prevalence weights were calculated for each VP in the VPop, the VPop’s weighted response to each intervention was calculated.

1. Continuous ACR-N responses were previously simulated for each VP in the cohort. Bin counts, which are natural for ACR20, 50, 70 data available in the literature from clinical trials, were employed in conjunction with the prevalence weights to assess clinical endpoints for the response to therapies.

2. Weighted means and standard deviations were also calculated for the VPop based on the simulated ACR-N responses for each VP. For the simulated ACR-N response sampled at time s to treatment t, the weighted mean and standard deviation were respectively calculated according to

(8) $${\bar{x}}_{t,s}=\sum_{i=1}^{N}w_{i}x_{i,t,s}$$

and

(9) $$s_{t,s}=\sqrt{\frac{N\sum_{i=1}^{N}w_{i}{\left(x_{i,t,s}-{\bar{x}}_{t,s}\right)}^{2}}{N-1}}$$

Here, x_i,t,s_ is the response for VP i to treatment t sampled at time s and N is the number of VPs used in the calibration. The population ACR 20, 50, and 70 responses were also calculated by summing the total prevalence weight for VPs with clinical responses falling within each response bin.

### MAPEL step 4: agreement with clinical trial data and objective function evaluation

In order to assess the validity of the calculated VPop responses and ascertain a composite goodness-of-fit, a comparison was made to trial data reported in the literature. Note that in addition to matching the binned (e.g. < ARC20, ACR 20–50, etc.) ACR response, we calculate a trial mean and standard deviation from the literature data. The mean and standard deviation for trial data were respectively calculated from the reported distribution according to

(10) $${\bar{x}}_{\mathrm{Tt},s}=\frac{1}{N_{\mathrm{Tt},s}}\sum_{b=1}^{B}c_{b}n_{\mathrm{Tt},s,b}$$

and

(11) $$s_{\mathrm{Tt},s}=\sqrt{\sum_{b=1}^{B}\frac{n_{\mathrm{Tt},s,b}}{N_{\mathrm{Tt},s,b}-1}{\left(c_{b}-{\bar{x}}_{\mathrm{Tt},s}\right)}^{2}}$$

Here, B is the number of ACR response bins, N_T,t,s_ is the number of trial patients for therapy t sampled at time s, n_Tt,s,b_ is the number of patients in trial response bin b for therapy t sampled at time s, and c_b_ is the midpoint of response bin b (e.g. 35 for ACR20-50). The trial mean and standard deviation were included in the optimization to avoid situations where a good fit for a VPop the distribution could be achieved by shifting the weights to the VPs that fell at, e.g. the upper, edge of each response bin. The comparisons to clinical data for the population mean, standard deviation, and bin distribution were made by calculating p-values with the t-test, f-test, and chi-squared test, respectively. Fisher’s method was used to calculate the composite goodness-of-fit from individual p-values for each trial statistic to be matched. The sum of logarithms of the individual p-values was used as the optimization objective. This choice for an objective function ensured the p-values for individual statistical tests converged well and was also consistent with the application of Fisher’s method for aggregating multiple p-values together for the composite goodness-of-fit. R’s Nelder-Mead optimization algorithm was employed to update the axes weights and iterate until convergence.

### Additional algorithmic details

For the purpose of optimization, bin probabilities were converted using a hyperspherical transform. The transform enabled the optimization’s independent variables to vary over an infinite range and avoided edge complications during convergence. To develop alternate VPops and explore variability in optimal solutions while maintaining statistical agreement with clinical trial results, valid VPops were used as seeds for randomization of axes weights and the development of new populations. The MAPEL algorithm, employed targets, VP cohort, and script for running a test case are supplied in Additional file 2.

### Sensitivity analysis

We performed an analysis of the pathway effects of IFNβ on the response to rituximab therapy. IFNβ effects were either applied in a “one-on” or “one-off” fashion, similar to the one-off method applied previously [18]. After virtual patients stabilized on methotrexate therapy in the biosimulation, either one IFNβ pathway effect was locked (“off”) or only one IFNβ pathway effect was unlocked (“on”) and able to change in response to alteration in IFNβ concentrations caused by the application of rituximab. The simulated ACR-N response was measured, and the previously determined prevalence weights for the alternate populations were applied to calculate each VPop’s response.

### Biomarker analysis

After developing alternate VPop prevalence weight solutions, an analysis of biomarkers was conducted. Measurements of synovial mediator concentrations were made at baseline, after twelve months of simulated methotrexate treatment, and prior to the addition of the biological therapy. One mediator was selected from sets of highly correlated mediators (ρ > 0.8), retaining the mediator with the lowest average correlation with other synovial mediators. For each virtual population, the best linear regression models were identified using an exhaustive search with the LEAPS package [28] in R. An initial analysis of the Bayesian information criterion (BIC, a widely used model selection metric that combines the explained variance and model complexity) for a subset of VPops suggested employing a model of 27 regressors. Alternatively, an analysis of adjusted R^2^ for each model size found a model size of five analytes yielded an adjusted R^2^ of 0.75. The purpose of the analysis was to extract the most informative analytes, and overfitting the model was a substantial concern. Five analytes presented a reasonable tradeoff between model complexity and ability to describe the virtual population. We present the results for the adjusted R^2^ for linear models constructed for each VPop as a function of model size in Additional file 3.

## Results and discussion

### Calibration of the ensemble of virtual populations

The MAPEL algorithm links VP mechanistic axes to population-level statistics reported in clinical trials (Figure 2). When calibrating the clinical response of the VPops, we elected to focus on ACR results due to the wealth of data available from clinical trials in patients that do not respond well to methotrexate and the continued interest in this population for the development of new therapies. The unweighted cohort VPs exhibited a full spectrum of simulated ACR-N responses to all of the simulated interventions (available in Additional file 2), from no improvement (0%) to marked improvement (87%) for all tested therapies. Slightly larger ranges were covered with some of the interventions (21% worsening to 100% improvement), but remained within the feasible range in Additional file 1.

**Figure 4:**
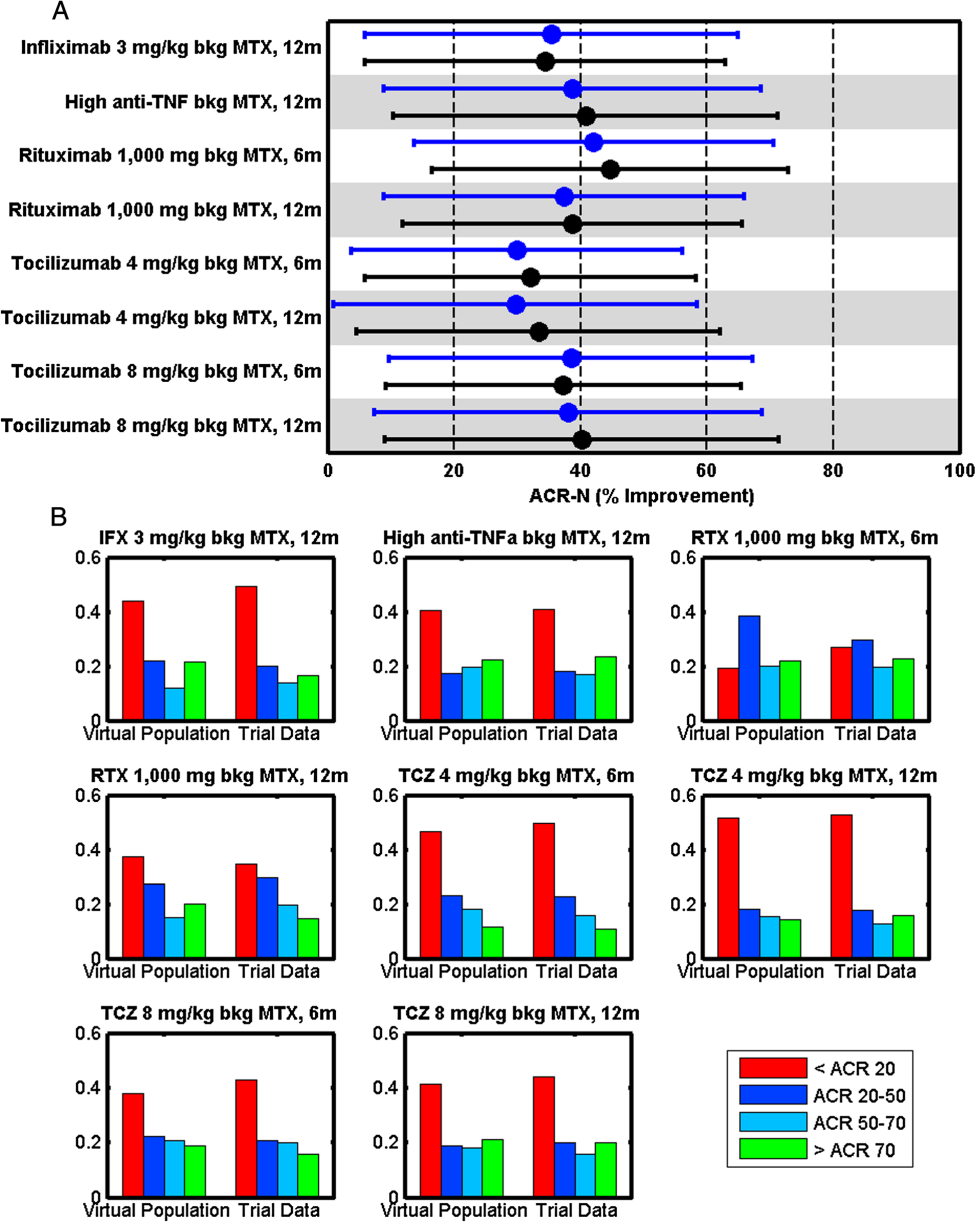
**Calibration result for a single virtual population.** As described in the methods, each VPop is assessed for agreement with mean, standard deviation, and binned distribution results from clinical trial data on the basis of ACR scores. (**A**) The mean (dot) and standard deviation (error bar) of low-dose anti-TNF, high-dose anti-TNF therapies (infliximab, adalimumab), rituximab, and tocilizumab was calibrated to clinical trial results. (**B**) The ACR distribution was also calibrated to clinical trial data. The y-axis indicates the fraction in the bin. Note that every virtual population had to compare well against these criteria, with a composite goodness-of-fit p-value greater than 0.05, to be included in subsequent analyses.

The MAPEL algorithm was successfully employed to build alternate VPops that matched trial data with a composite goodness-of-fit p-value greater than 0.05. A sample calibration result for a single VPop is presented in Figure 4. In total, 768 alternate VPops were created that statistically agree with ACR-N responses derived from clinical trials for approved therapies with a composite goodness-of-fit greater than 0.05. Notably, the 768 alternate VPops that form the basis for analyses were informed by all 1,206 VPs from the cohort. That is, both VPs that ultimately receive higher prevalence weightings and VPs that ultimately received numerically small weights were included to optimize the match with clinical results for each VPop through the axes weights.

**Figure 5:**
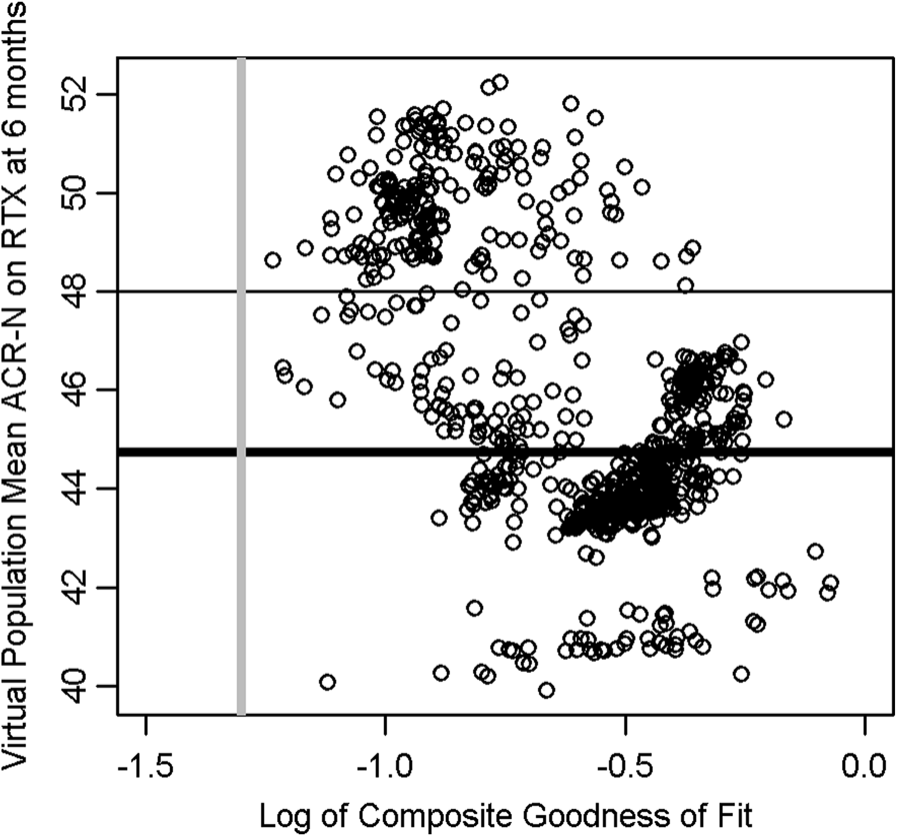
**Virtual populations were selected with composite goodness-of-fit p-values greater than 0.05.** VPops developed with the MAPEL algorithm give good agreement with clinical trial results as assessed by the composite goodness-of-fit criterion. Each circle represents a VPop, and smaller p-values (x-axis) imply a worse goodness-of-fit. MAPEL results were filtered for VPops with a composite goodness-of-fit greater than 0.05 and used for further analyses (vertical grey line). Of particular interest was the response to treatment with rituximab (y-axis), and the reported mean clinical response at 6 months is shown by the heavy black line. To explore the mechanistic characteristics of populations that respond well, VPops with an ACR-N response greater than 48 were contrasted with VPops with responses less than the mean.

The composite goodness-of-fit for the full ensemble of acceptable VPops is plotted against the response to rituximab (RTX) at 6 months in Figure 5. MAPEL generated VPops with markedly different mean responses to rituximab from the same virtual patient cohort while maintaining the good composite fit. Note that we elected to compare simulation results to the ritxuximab trial by Edwards et al. when calibrating the virtual populations [22]. Data from the larger SERENE trial [29] were available, but the study protocol allowed for an optional second injection of rituximab after 24 weeks. Since it was important to ensure the virtual patients were all on the same protocol in the simulation and we wanted to ensure the proper response kinetics over a full year, we did not aggregate the SERENE data for comparison.

### Mechanistic alterations in pathophysiology govern the response to rituximab

**Figure 6:**
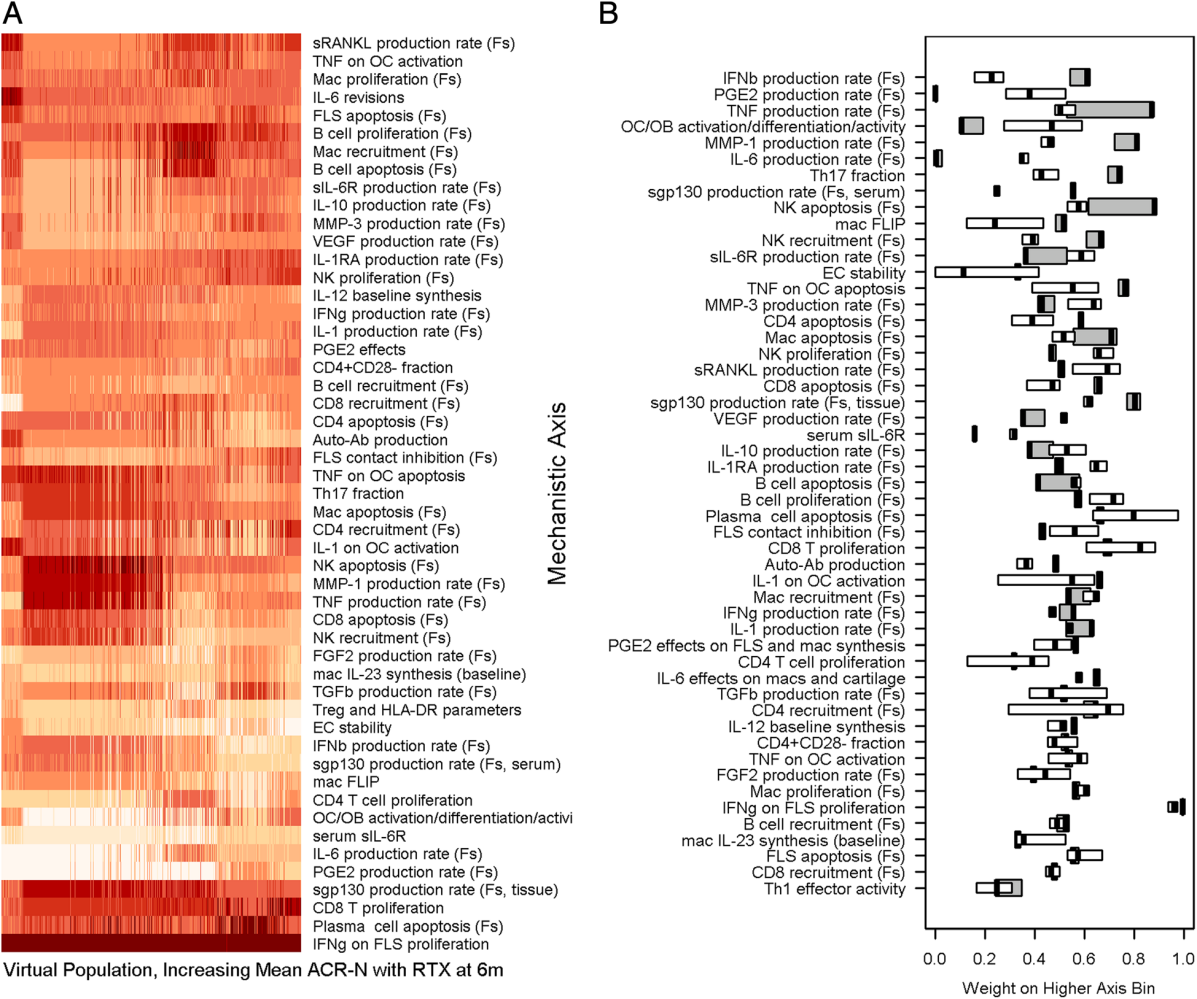
**Alternate virtual populations exhibit distinct mechanistic patterns that distinguish populations that respond well to rituximab.** (**A**) Mechanistic differences in the VPops were apparent from a heatmap analysis. VPops were ordered by increasing ACR-N response to rituximab. The color indicates the weight on the higher axis bin, since each axis was split into two bins for assigning weights with MAPEL. Darker red implies a higher weight on the higher-valued bin, meaning the activity governed by the axis is generally high in the VPop. (**B**) Differences were apparent between VPops with greater responses to rituximab and those with less than the mean response. VPops with a mean ACR-N response greater than 48 (white boxes) were contrasted with virtual populations with less than the observed clinical mean response (grey boxes). Interquartile ranges are also depicted by the box widths. The heavy black line shows the median. Mechanistic axes are ordered from the largest difference between the medians at the top of the figure to least differentiated at the bottom of the figure.

We performed a heat map analysis to interpret the mechanistic differences underlying the pathophysiology in VPops that responded well to rituximab therapy. Since each mechanistic axis was divided into two bins, the weights for the higher bin are shown in Figure 6A. The VPops were ordered along the x-axis by increasing mean response to rituximab. The heat map illustrates population variation across all mechanistic axes, with the possible exception of the effect of IFNγ on FLS proliferation. The result is interesting because there are conflicting reports in the literature regarding the ability of IFNγ to induce FLS proliferation [30-32], and the axis was designed to allow for potentiation of proliferation (low values) or inhibition of proliferation (high values). The VP cohort included patients with axes coefficients that represented at least 92% of the pre-determined feasible biological range (axes coefficients for the cohort included in Additional file 2), so conclusions drawn are robust to alterations in the underlying pathophysiology. The result gives systems-level support to the hypothesis that IFNγ predominantly inhibits FLS proliferation in vivo. Furthermore, no clear trend for therapeutic response is observed in a single mechanistic axis, in agreement with the polyfactorial nature of RA.

To more completely investigate the mechanistic contrasts between VPops that responded well to rituximab from those that responded poorly, we grouped VPops with larger mean responses (ACR-N > 48, thin line in Figure 4) and compared them to VPops with smaller mean responses (ACR-N < 44.75, heavy line in Figure 4). The cutoff of 44.75 was chosen since this was the mean response calculated from in the rituximab trial [22]. Rather than directly contrast populations above and below the mean, we noticed a sizable number of alternate VPops with a mean ACR-N score greater than 48. These VPops appeared to incorporate substantial mechanistic heterogeneity (Figure 6A, populations at right). To contrast these VPops, boxplots illustrating the median and interquartile range of the axes coefficients for these groups were constructed (Figure 6B). The axes were sorted by decreasing differences in the median upper axis bin weight. The axes in Figure 6B are ordered vertically, with decreasing separation between the VPops with strong and weaker responses. The axes near the top of the list exhibit the most separation between VPops that exhibit strong improvements with rituximab and those that do not. Interestingly, IFNβ production was the leading mechanistic axis identified, with lower IFNβ production associated with better response to rituximab.

Figure 6B is in agreement with published reports suggesting that a low type I interferon signature at baseline predicts responsiveness to rituximab [19]. Although the authors speculated that the reasons could be a less B cell-dependent phenotype, they also suggest this explanation seems unlikely since all patients in their study were rheumatoid factor positive and/or anti-citrullinated peptide antibody positive. Furthermore, the non-response to rituximab appears to be associated with the persistence of B cells [33,34]. We included alterations in B cell pathology in our axes and therefore sought to contrast the role the axes played in the response to rituximab. We “flipped” each mechanistic axis value for VPs weighted heavily in the population that responded well to values representative of populations that responded poorly to RTX (Additional file 4). The analysis confirmed a quantitatively important mechanistic effect for IFNβ production in the response to RTX. Interestingly, the axis flip analysis predicted that FLS parameters, more than directly modulating B cell growth, recruitment, or apoptosis, play a role in the inflammatory response to a therapeutic that depletes the B-cell population. The result is consistent with the view that that patient-to-patient variation in FLS biology may influence response to rituximab. The result is also consistent with the observation that the transplant of IFNβ competent FLS suppress inflammation in a collagen-induced arthritis model in IFNβ deficient mice [35].

### IFNβ effect sensitivity analysis

**Figure 7:**
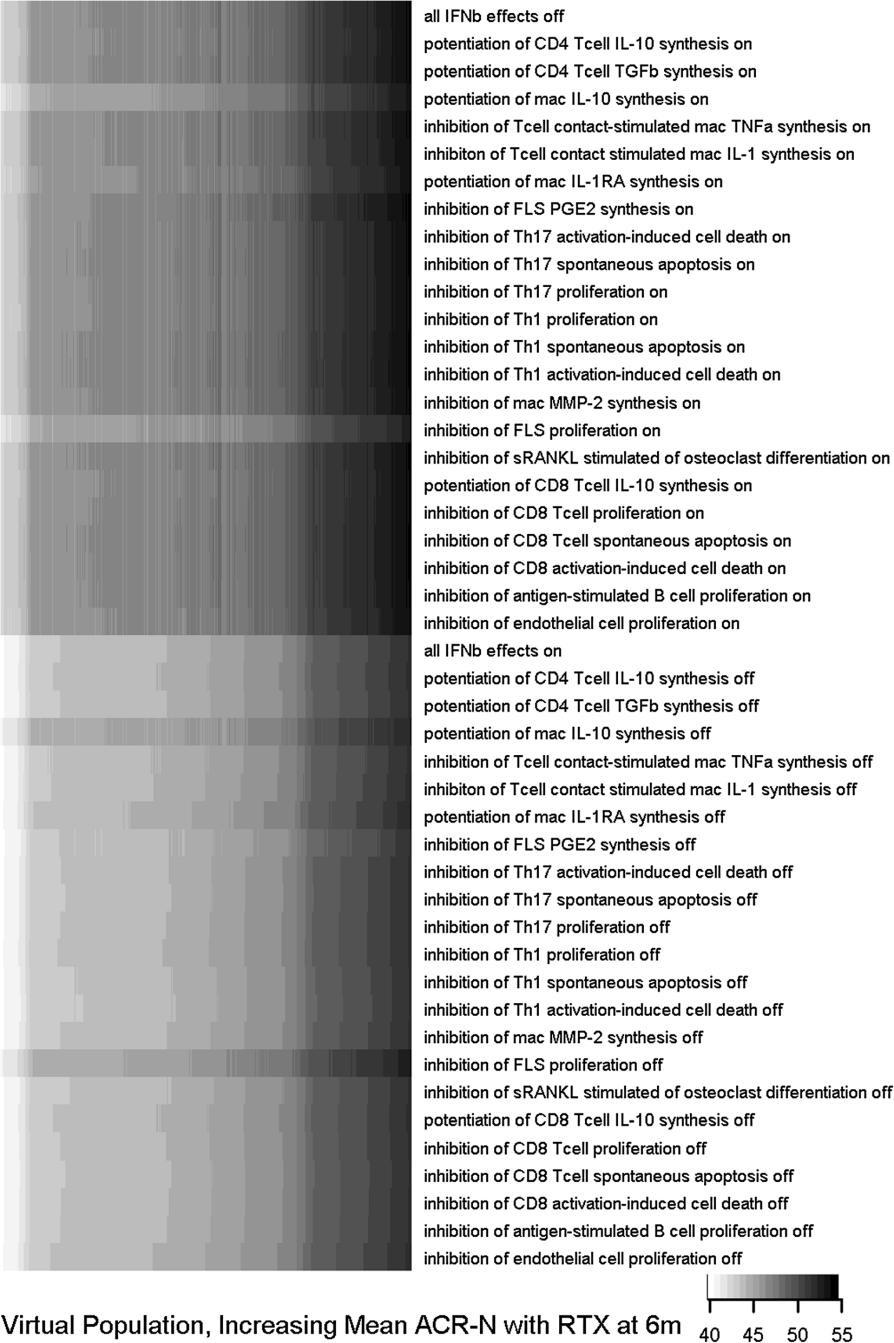
**Sensitivity analysis in the ensemble of populations proposes IFNβ-mediated mechanisms that dominate the clinical response to rituximab.** Sensitivity analysis was performed by altering the indicated IFNβ-effect in the biosimulation for each VP while administering rituximab, re-running simulations for the entire cohort, and recalculating population-level statistics using the previously calculated axes weight solutions from MAPEL. The populations are ordered along the x-axis by increasing response to rituximab, and the shade indicates the mean ACR-N score for the VP cohort. In the top series, one effect of IFNβ was allowed to respond to the application of rituximab. In the bottom series, one effect of IFNβ was held fixed during the response to the application of rituximab. VPops were primarily sensitive to the effects on IFNβ on macrophage IL-10 synthesis and FLS proliferation.

To develop mechanistic insight into the role of IFNβ in the response to rituximab, we conducted a sensitivity analysis within the VPops (Figure 7). Across the ensemble of VPops, we observed the response to rituximab to be sensitive to the effects of IFNβ on macrophage IL-10 synthesis as well as FLS proliferation. It has been noted that IFNβ can potentiate IL-10 synthesis by macrophages, which itself has general anti-inflammatory effects [36-38]. Furthermore, IFNβ has been noted to inhibit FLS proliferation [39-41]. The results suggest decreases in IFNβ partially counteract the response to rituximab through secondary effects in the cytokine signalling network. Turning off the effect of therapy on either of these two pathways strengthened the response to rituximab (potentiation of mac IL-10 synthesis off, inhibition of FLS proliferation off, Figure 7). Because the most sensitive effectors of IFNβ were primarily inhibitory, removal of the effects of rituximab on IFNβ resulted in an improvement in ACR-N score. The effect of turning IFNβ effects off is a consistent improvement in response across the entire ensemble of 768 statistically calibrated VPops at 6 months, despite the presence of some cohort VPs that have a decrease in ACR-N response to rituximab with the IFNβ effects turned off (290 of 1,206 VPs, not shown). The consistency of the VPop analysis illustrates the advantage of employing the MAPEL algorithm to perform analyses that are statistically powered at the population level and more robust to the underlying pathophysiology of individual VPs. Interestingly, induction of type I interferon activity has been observed to correlate with the improvement on rituximab [42], in agreement with a putative anti-inflammatory role for type I interferons in RA. An anti-inflammatory role for type I interferons in RA is consistent with our findings for synovial IFNβ in the VPops. This does not contradict the finding that a low type I interferon signature prior to the initiation of therapy predicts the response to rituximab [19], as these patients may have higher synovial inflammation at baseline.

Other mechanistic axes that appear to also be prognostic of the response to rituximab include prostaglandin E2 (PGE2), TNF, and IL-6 production (Figure 6B). PGE2 is a pleiotropic cytokine with both pro- and anti- inflammatory effects. For example, PGE2 inhibits the production of the potent pro-inflammatory cytokine TNF by macrophages [43-45]. By contrast, PGE2 can also potentiate pro-inflammatory Th17 T cell responses through IL-23 [46,47]. It would be of interest to note whether there is an association between NSAID (non-steroidal anti-inflammatory drug) use and response to rituximab and, perhaps more importantly, whether NSAIDs may enhance the response to rituximab. Combining NSAIDs with rituximab in our simulations resulted in variable changes in ACR-N responses (−6% to 12%) in the VPops compared to rituximab alone (Additional file 5), in agreement with the general ineffectiveness of NSAIDs in RA. However, there were some VPs that did receive a marked benefit from the combination (weightings not shown), suggesting that targeting a subpopulation of such patients may be a future therapeutic direction. TNF and IL-6 are well established and validated clinical targets for RA for which approved biologic therapies are available. OC/OB activity and MMP-1 also appear to be good markers, suggesting that feedback from the bone and cartilage compartments may provide a mechanism to influence response to rituximab.

### Formulation and analysis of multianalyte biomarkers predictive of the response to rituximab

**Figure 8:**
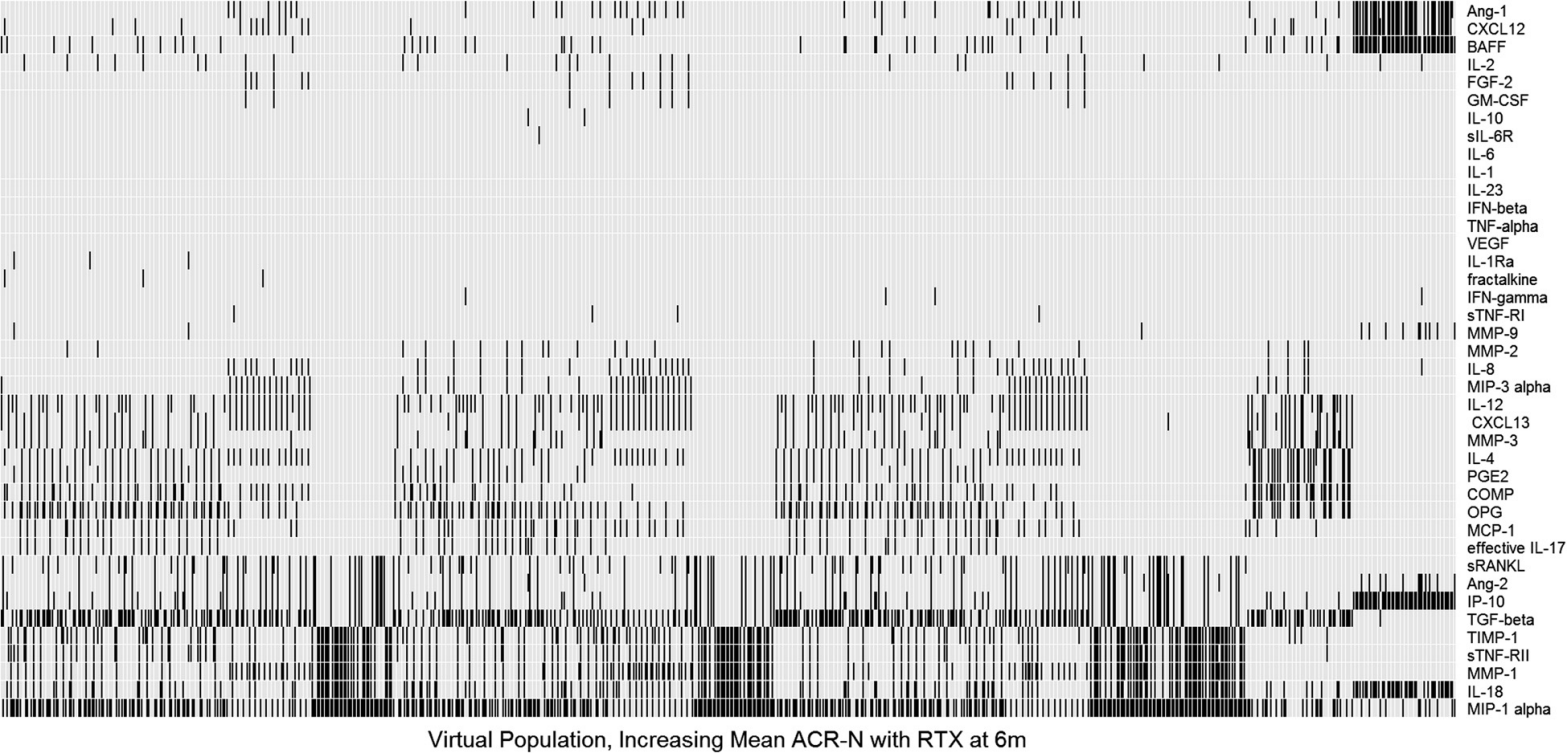
**Biomarker analysis in virtual populations robustly identifies synovial mediators that predict response to rituximab.** An exhaustive linear regression with five regressors was performed with each VPop to identify the five synovial analytes most predictive of the response to rituximab.

To extend our analysis to markers that could possibly be measured in the serum of RA patients, multivariate linear regression was used to identify baseline synovial mediators most predictive of the response to rituximab. Validation resulted in the selection of a model with 5 regressors to yield an adjusted R^2^ of 0.75 (Additional file 3). The selected markers for each VPop are shown in Figure 8, and their frequencies across VPops are summarized in Additional file 6. Although mechanistically implicated by both the differences in axis weight and axis flip experiments, IFNβ was not predicted to be a good marker as assessed by synovial mediator concentrations. The result illustrates characteristics of therapeutic response pathways that may be uncovered with different bases (i.e. analysis of model inputs (mechanistic axes) versus model outputs (synovial mediators)). The mechanistic axes are representative of the underlying propensity of cells to produce IFNβ, e.g. the propensity for underlying transcription and translation of the gene. However, markers of downstream effects of IFNβ may better correlate with the clinical response than the IFNβ concentration. Reinforcing the link between FLS and B cells, soluble factors produced by FLS, such as MIP-1α, TGFβ, and MMP-1 were frequently amongst the best markers of response to rituximab. Admittedly, FLS are capable of synthesizing many inflammatory mediators. However, it is striking that in addition to predicting structural endpoints, MMPs may also serve as predictors of inflammatory responses, likely due to their association with FLS activation. We also observed markers associated with B cell activity, including B cell activating factor (BAFF), as well as a chemoattractant for B cells and other lymphocytes, CXCL12. BAFF has been implicated as playing a role in B cell activation and may contribute to disease progression in RA via autoantibody production [48-50].

### Summary of advantages of MAPEL

There are a number of advantages to using MAPEL in order to develop VPops. First, all VPs are utilized to develop each VPop. Since all VPs are defined by the mechanistic axes, even those that ultimately receive low weights inform the analysis. For example, VPs with low weights may impact the axes weights if, during the convergence process, attempting to increase their weight decreases the composite goodness-of-fit. Second, the method allows the rapid generation of alternate VPops that are consistent with clinical data to enable population-level hypothesis generation while considering the goodness-of-fit. Third, the method is scalable to include many VPs. Because the weights are calculated from the axes, rather than the VPs, a large number of diverse VPs can be included. For example, we included 1,206 VPs when generating 768 alternate VPops. In the study discussed previously, 1 VPop was created with 145 VPs [14]. A large degree of mechanistic variability can be introduced into the VPs to facilitate exploratory analyses of the effects of mechanistic variability. We varied 51 mechanistic axes, compared with, for example, six or eleven parameters in a model of DILI [15,16].

### Additional applications of MAPEL

Although we focus on the application of MAPEL to biosimulation results, there are additional potential applications that may find utility for researchers engaged in other systems biology and bioinformatics research. In theory, MAPEL can be applied with any high-dimensional dataset matched with clinical outcomes to extrapolate prevalence in a larger population. For example, plasma metabolite concentrations, tissue cytokine levels, proteomic measures, or other high-throughput data sources could be implemented as the mechanistic axes. Methods such as principal component analysis (PCA) or functional groupings could serve to condense the effective dimensionality of the axes if the dimensionality becomes intractable. Although the approach would require sample-matched results from multiple interventions in the small dataset, MAPEL would then essentially serve as an advanced regression technique that could be used to propose mechanistic prevalences in populations from larger trials. Furthermore, although we have employed the Entelos RA PhysioLab® platform, other mechanistic modeling approaches can be utilized with MAPEL to develop phenotype-informed weights for model solutions.

## Conclusions

The MAPEL algorithm integrates mechanistic biosimulation results with clinical trial statistics to enable statistically-validated analyses of biomarkers and mechanistic determinants of disease progression at the population level. Applying weights directly to the axes, rather than to individual VPs, is a novel approach with several advantages. First, the algorithm executes with reasonable computational speed (less than 10 minutes per population on a Core 2 Duo processor for the cases explored here). In contrast to developing independent weights for individual virtual patients, the number of parameters is reduced and large cohorts of thousands of virtual patients become tractable for analysis. Second, the axes themselves describe biologically relevant mechanisms that yield insights into treatment response consistent with the literature for known therapeutics. The mechanistic axes complement synovial outputs to give a more complete understanding of the pathophysiology. Finally, integration with a mechanistic biosimulation platform facilitates testing at the population level to better segregate mechanistic factors from those merely arising from association. The MAPEL algorithm is a significant advance towards in the development of tools to enable a systems-level understanding of complicated diseases, such as RA.

We illustrate how these methods can be applied to interpret the type I interferon signature that has been observed to vary with patient responses to rituximab. Together, our results suggest two distinct mechanisms may play a role in establishing the poor response to rituximab. First, type I interferons such as IFNβ may play some role to reduce inflammation in RA. In patients with an underlying pathophysiology that is less driven by B cells and FLS, type I interferon reduction by rituximab may counteract some of the therapeutic effect. Second, in patients with pathophysiologies where B cells play a quantitatively more substantial role, the suppressive effects of the type I interferons are still present but play a smaller role. The pathology is substantially altered to facilitate a clinically adequate response.

## Competing interests

JRC and TP are currently salaried employees of Entelos Holding Corp. BJS and FPC were salaried employees of Entelos, Inc. within the last five years. Entelos Holding Corp. is financing the manuscript, including the article-processing charge.

## Authors’ contributions

BJS contributed to the development and application of the MAPEL algorithm, designed the simulations for sensitivity analysis, performed the analyses and helped to draft the manuscript. FPC designed, developed, and applied the MAPEL algorithm, and helped to draft the manuscript. TP conceived and designed the MAPEL algorithm and helped to draft the manuscript. JRC conceived of the study, lead in its design and coordination, developed the virtual patient cohort, and helped to draft the manuscript. All authors read and approved the final manuscript.

## Supplementary Material

Additional file 1 **Acceptance ranges for biosimulation results that define feasible virtual patients to include in the cohort.** The literature was reviewed for the density of inflammatory cells found in the synovial tissue and cytokine concentrations measured in the serum. Click here for file

Additional file 2 **MAPEL archive.** The archive contains: Virtual patient cohort - The spreadsheet includes the measures used to run the MAPEL algorithm. MAPEL - The MAPEL algorithm is run in the R environment. MAPEL bin targets - Discrete distribution response targets were calculated from published clinical trial data and used to guide the MAPEL algorithm. MAPEL mnsd targets - Mean and standard deviation targets were calculated from published clinical trial data and used to guide the MAPEL algorithm. MAPEL utilities - Utilities used for running the MAPEL algorithm. MAPEL packages - Install packages needed to run MAPEL algorithm. Sample MAPEL script - Simple script for running the MAPEL algorithm using the supplied virtual patient cohort and targets. Click here for file

Additional file 3 **Selection of biomarker regression model size.** For each set of VPop weights, exhaustive multivariate linear regression was performed to identify the best model for each model size. The adjusted R^2^ was calculated for the best model of each size for each VPop. The black line indicates the mean and the red lines indicate the range observed in the VPops. Five regressors provided an adjusted R^2^ of 0.75; an increase to 10 regressors only improved the adjusted R^2^ to 0.82. Click here for file

Additional file 4 **Axis flip experiments.** Axis flip experiments were performed to distinguish mechanistically consequential alterations in the mechanistic axes in VPops that responded well to rituximab. This file contains additional methodological details, results, and additional discussion. Click here for file

Additional file 5 **Effect of NSAIDs on the response to rituximab at 12 months.** VPs were maintained on background methotrexate therapy, and treated with either NSAIDs, rituximab, or combination therapy. The response at 12 months was assessed and is indicated by the color bar (VPops are ordered by their response to rituximab at 6 months, which expectedly correlated well with the response at 12 months). Some VPops exhibited an average ACR-N benefit of up to 12% from the combination, especially those that tended to respond poorly to rituximab alone. However, some VPops also exhibited a mean decrease relative to rituximab of about 6%. Click here for file

Additional file 6 **Frequency of occurrence of synovial mediators amongst the best five regressors for the alternate virtual populations.** Multivariate linear regression was used to identify baseline synovial mediators most predictive of the response to rituximab. Click here for file
