# Supplementary figures and images for: Alternate virtual populations elucidate the type I interferon signature predictive of the response to rituximab in rheumatoid arthritis
Source: BMC Bioinformatics. 2013 Jul 10;14:221. doi: 10.1186/1471-2105-14-221 (PMC3717130; doi:10.1186/1471-2105-14-221)

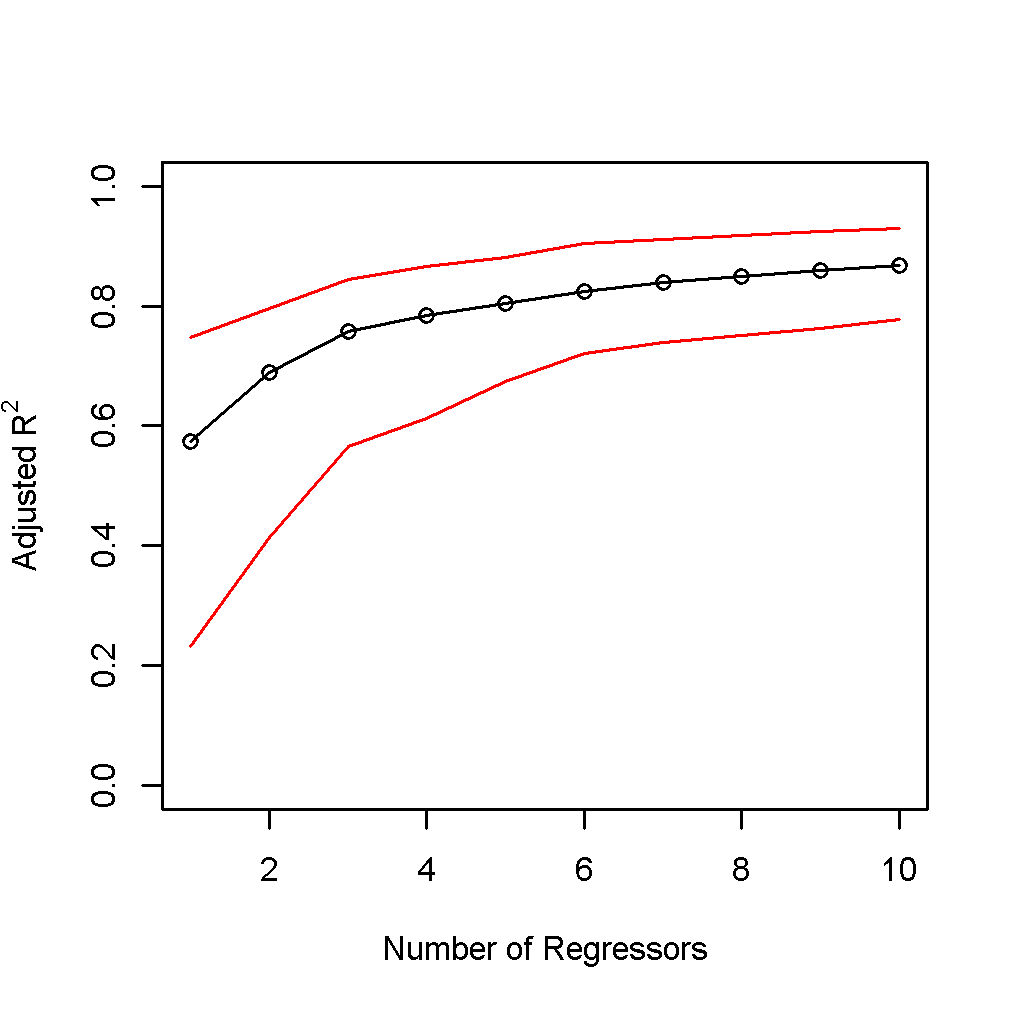

Supplement: Additional file 3 — Selection of biomarker regression model size. For each set of VPop weights, exhaustive multivariate linear regression was performed to identify the best model for each model size. The adjusted R2 was calculated for the best model of each size for each VPop. The black line indicates the mean and the red lines indicate the range observed in the VPops. Five regressors provided an adjusted R2 of 0.75; an increase to 10 regressors only improved the adjusted R2 to 0.82. [file 1471-2105-14-221-S3.tiff]

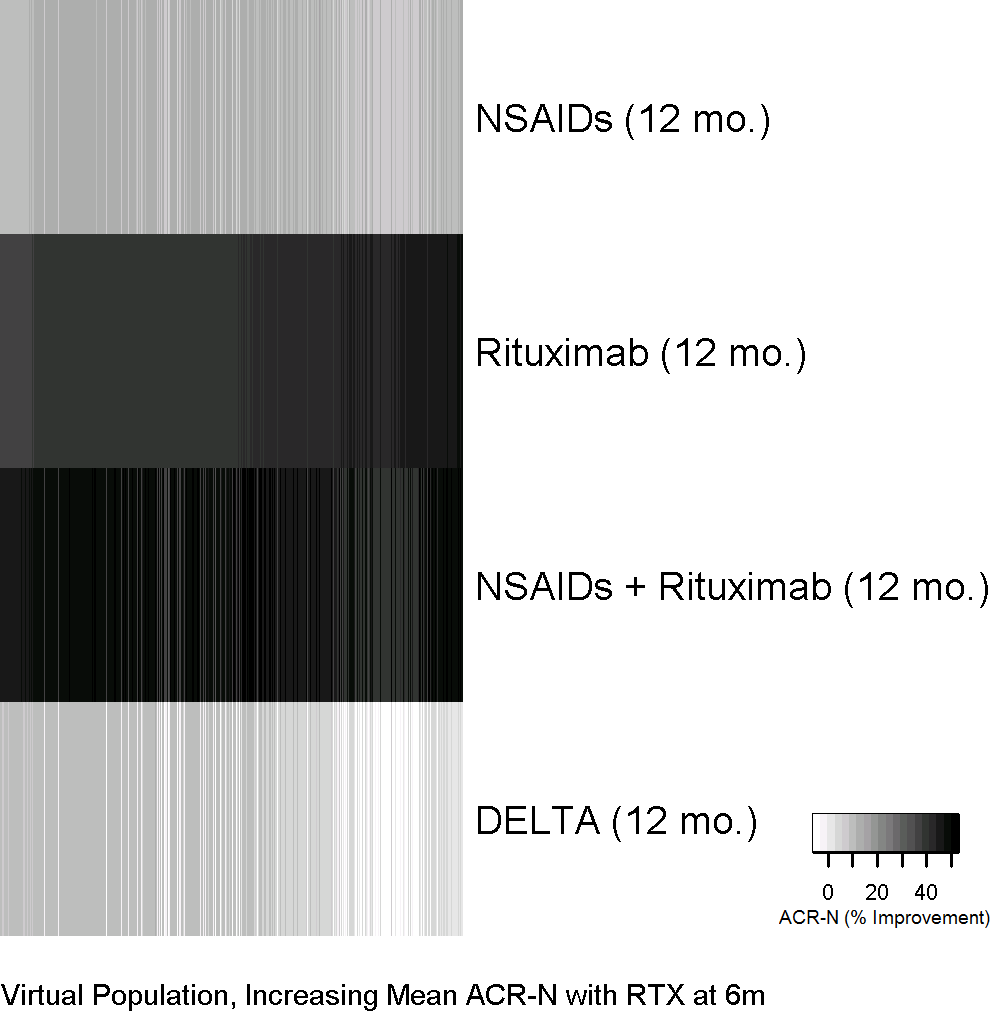

Supplement: Additional file 5 — Effect of NSAIDs on the response to rituximab at 12 months. VPs were maintained on background methotrexate therapy, and treated with either NSAIDs, rituximab, or combination therapy. The response at 12 months was assessed and is indicated by the color bar (VPops are ordered by their response to rituximab at 6 months, which expectedly correlated well with the response at 12 months). Some VPops exhibited an average ACR-N benefit of up to 12% from the combination, especially those that tended to respond poorly to rituximab alone. However, some VPops also exhibited a mean decrease relative to rituximab of about 6%. [file 1471-2105-14-221-S5.tiff]
