# Supplementary material for: Alternate virtual populations elucidate the type I interferon signature predictive of the response to rituximab in rheumatoid arthritis
Source: BMC Bioinformatics. 2013 Jul 10;14:221. doi: 10.1186/1471-2105-14-221 (PMC3717130; doi:10.1186/1471-2105-14-221)
Supplement: Additional file 4 — Axis flip experiments. Axis flip experiments were performed to distinguish mechanistically consequential alterations in the mechanistic axes in VPops that responded well to rituximab. This file contains additional methodological details, results, and additional discussion. [file 1471-2105-14-221-S4.pdf]

Axis flip method

Axis flip experiments were conducted to delineate which axes were playing a role in the response to rituximab and which were merely associated with the mechanistic determinants. Highly weighted VPs amongst the VPops with strong average responses to ritxumimab were selected to represent at least 75% of an averaged population. A representative axis coefficient value for each axis for VPops that responded poorly to rituximab was also determined. VPops with mean responses less than the mean response reported clinically were identified, and the median coefficient for each axis was determined was determined from these VPops. The axes coefficients for highly weighted, individual VPs identified from the VPops that responded well were each flipped to the median values for the poorly-responding VPops. New responses for the average, strongly-responding VPop to rituximab were then calculated with each axis-flip alteration.

Axis flip result

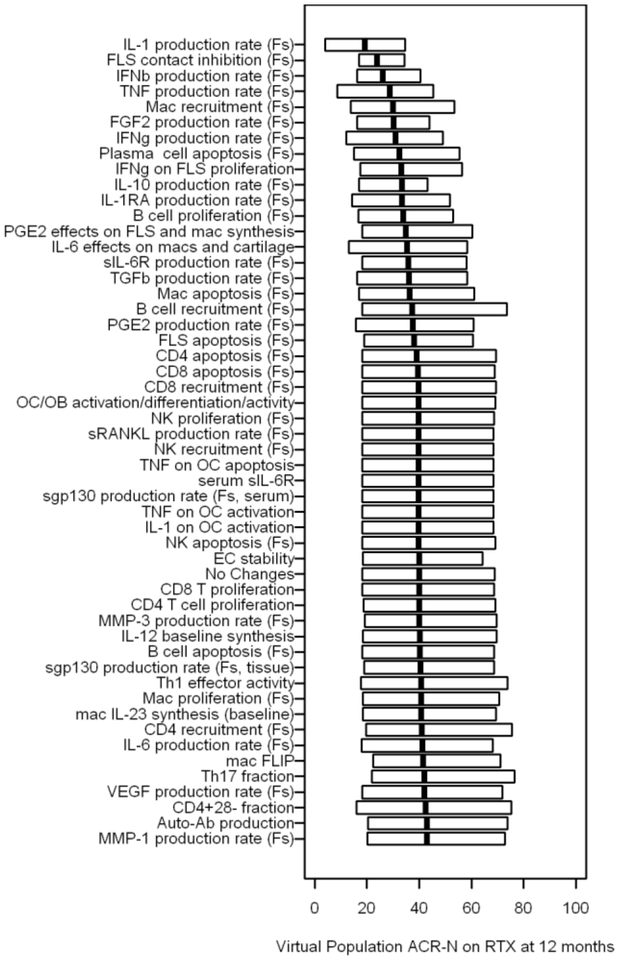

Additional file 4 Figure 1 – **Axis flip experiment results elucidate mechanistic axis markers with causative roles in the response to rituximab.**

The boxes depicted the interquartile range and the line depicts the mean. Axis near the have the largest effect to diminish the ACR response to rituximab when flipped to the value representative of the poorly-responding population.

Verification of mechanistic axes contributing to response by axis flip experiments

To better dissociate mechanistic marker axes that play a direct role in establishing the therapeutic response from those that might merely be associated with the mechanistic drivers, we performed axis flip experiments. The axes coefficients in weighted VPs that responded well to rituximab therapy were set equal to the median

value of weighted VPs that responded poorly. The analysis took advantage of the ability to perform biosimulation with the altered VPs, akin to the “one off” target evaluation method employed previously [1]. However, the axis flip analysis used population parameters derived from all VPs in the cohort and was therefore more robust to uncertainty in the underlying pathophysiology at the population level. As indicated in the figure, the axes near the top had the strongest individual influence in establishing the response to rituximab. At the top of the list was the production of another cytokine currently targeted by biologic therapies, IL-1. Contact inhibition of FLS was observed as the second most important mechanistic factor in this test. The result was interesting as a relationship between B cells and FLS has been previously postulated [2]. The authors speculated that TNF may be the link, and TNF production was also an axis that strongly influenced the response to rituximab.

References

1. Rullman J, Struemper H, Defranoux N, Ramanujan S, Meeuwisse C, van Elsan A: **Systems biology for battling rheumatoid arthritis: application of the Entelos PhysioLab platform**. *IEE Proc-Syst Biol* 2005, **152**:256–262.

2. Rosengren S, Kalunian KC, Kavanaugh A, Boyle DL: **CXCL13 as a marker for outcome of rheumatoid arthritis: comment on the article by Meeuwisse et al.** *Arthritis & rheum* 2011, **63**:3646–7.
